# Supplementary material for: Copper causes reduced nitrogen fixation but does not accumulate in the nodules of the legume Lotus japonicus
Source: PLoS One. 2026 May 8;21(5):e0349086. doi: 10.1371/journal.pone.0349086 (PMC13155547; doi:10.1371/journal.pone.0349086)
Supplement: S1 Table — (PDF) [file pone.0349086.s001.pdf]

Table 1. Nutrient solution recipe modified from Broughton and Dilworth [1].

Stock	Component	Amount in 500 mL stock	Stock added to 1 L H_2O (mL)	Concentration in nutrient solution
1	$CaCl_2 \bullet 2H_2O$	147.05 g	0.5	1 mM
2	KH_2PO_4	34.0 g	1	0.5 mM
3	K_2HPO_4	43.5 g	1	0.5 mM
4	Fe citrate	2.4 g	0.5	10 µM
5	$MgSO_4 \bullet 7H_2O$	61.65 g	0.5	250 µM
	K_2SO_4	43.5 g		250 µM
	$MnSO_4$	169 mg		1 µM
	H_3BO_3	123.5 mg		2 µM
	$ZnSO_4 \bullet 7H_2O$	143.8 mg		0.5 µM
	$CuSO_4 \bullet 5H_2O$	57.5 mg		0.2 µM
	$CoSO_4 \bullet 7H_2O$	28.0 mg		0.1 µM
	$NaMoO_4 \bullet 2H_2O$	24.0 mg		0.1 µM
6	KNO_3	50.5 g	0.5	0.5 µM

1. Broughton, WJ, Dilworth MJ. Control of leghaemoglobin synthesis in snake beans. Biochemical Journal. 1971;125(4):1075–1080. https://doi.org/10.1042/bj1251075.
