# Supplementary figures and images for: Copper causes reduced nitrogen fixation but does not accumulate in the nodules of the legume Lotus japonicus
Source: PLoS One. 2026 May 8;21(5):e0349086. doi: 10.1371/journal.pone.0349086 (PMC13155547; doi:10.1371/journal.pone.0349086)

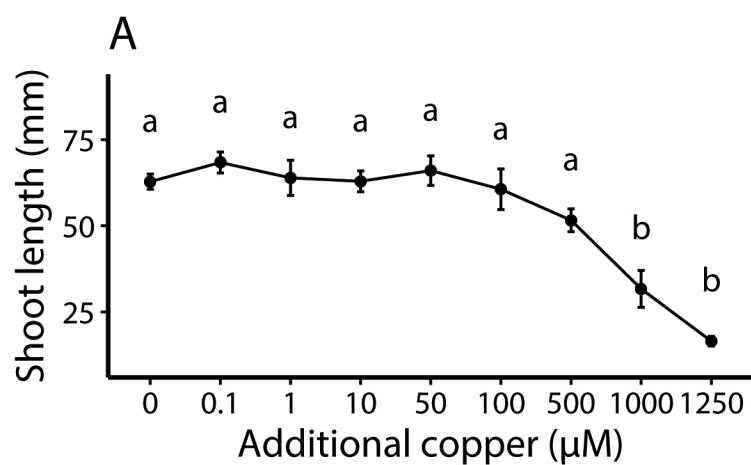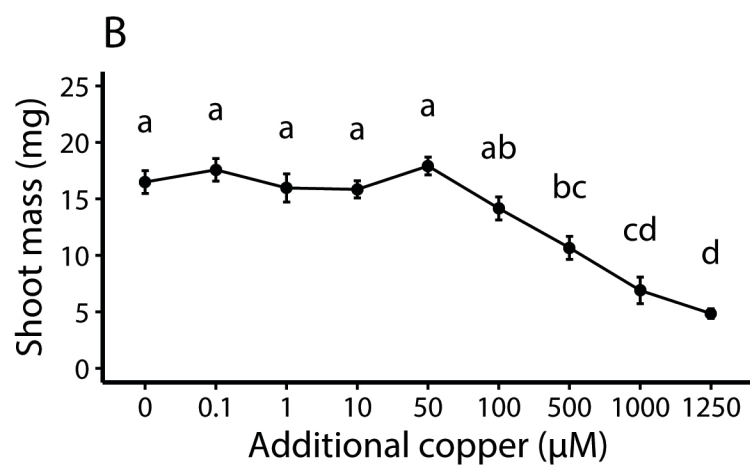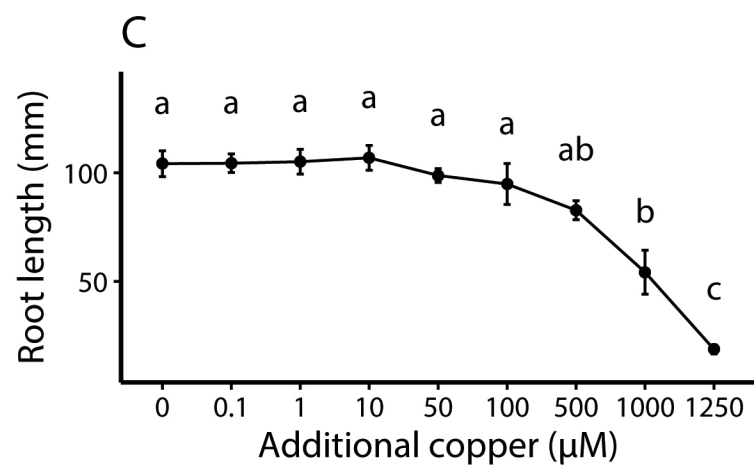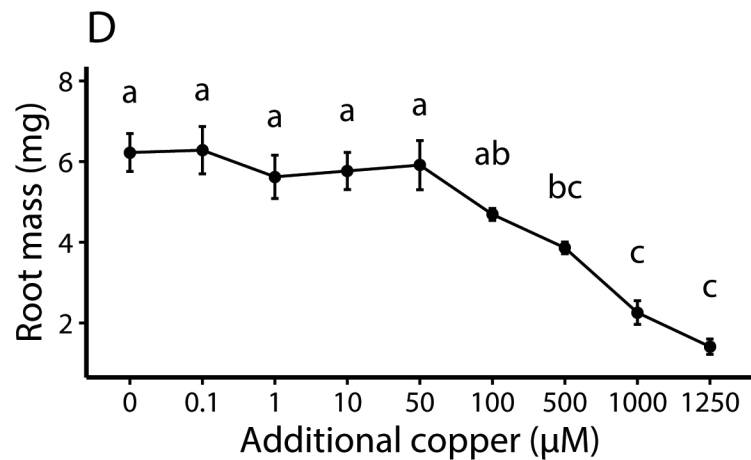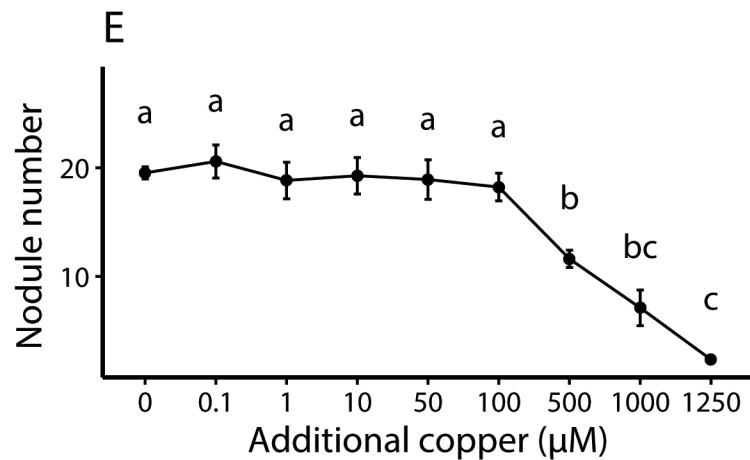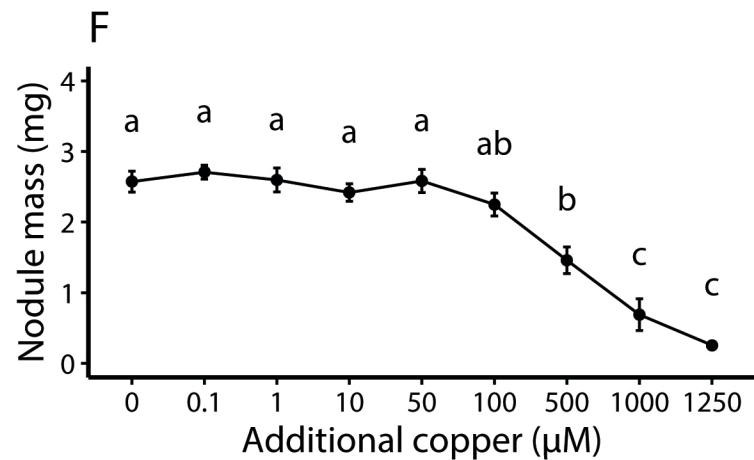

Supplement: S1 Fig — (A) Shoot length. (B) Shoot mass (mg). (C) Root length. (D) Root mass. (E) Number of nodules per plant. (F) Nodule mass. Error bars indicate SEM, n = 4 for all tissues. Letters indicate significant differences between groups (p < 0.05), analyzed with a one-way ANOVA followed by a Tukey’s HSD, with the exception of root mass data, which were analyzed with a Kruskal-Wallis test followed by Dunn’s pairwise comparisons because the data failed the homogeneity of variance test. (PDF) [file pone.0349086.s002.pdf]

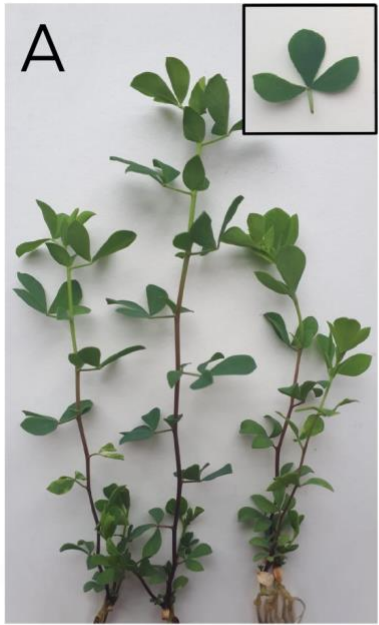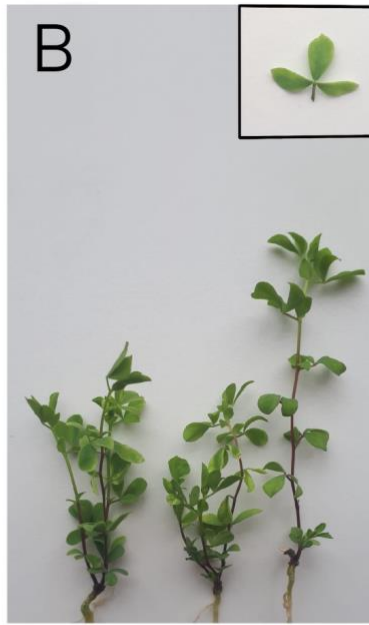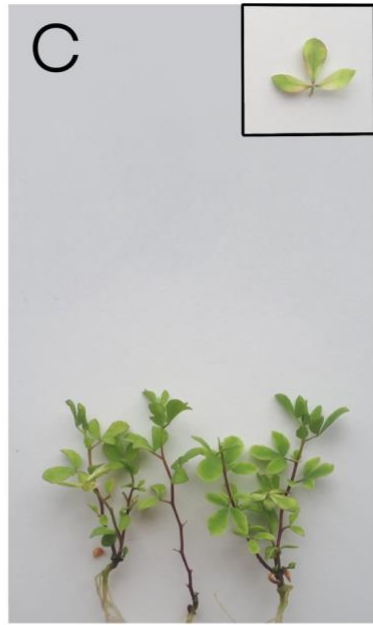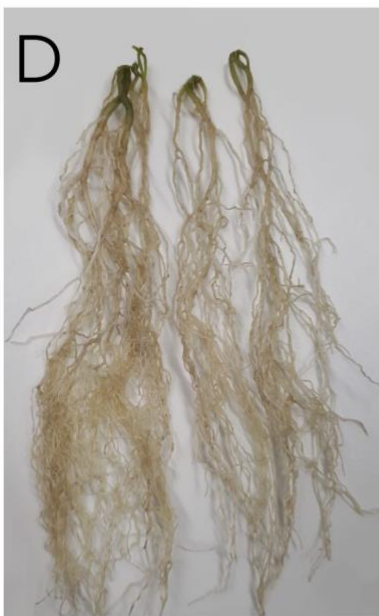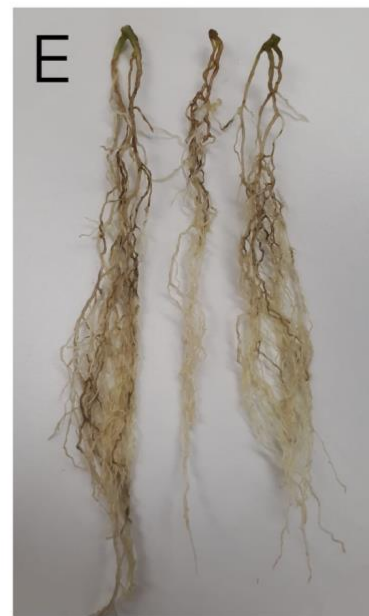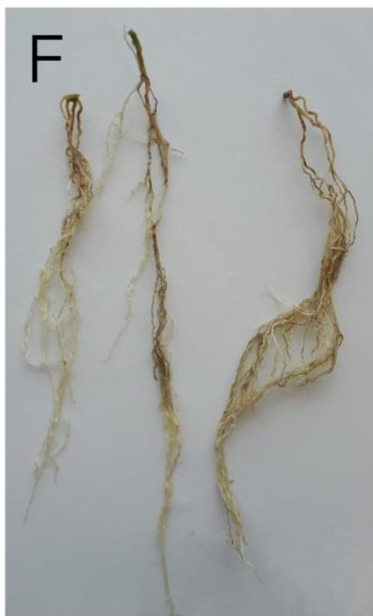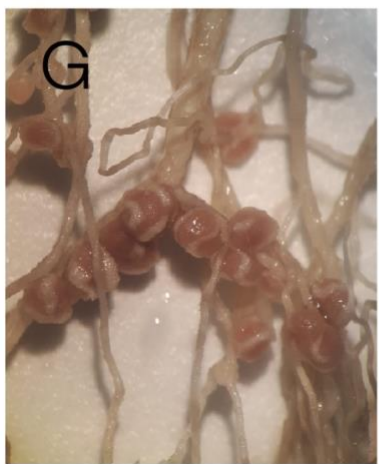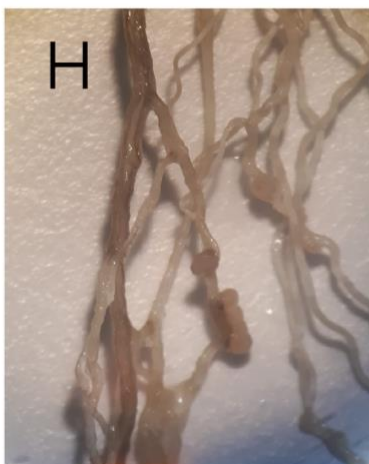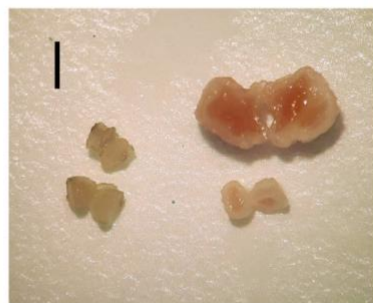

Supplement: S2 Fig — (A – C) Whole shoots, with a single trifoliate leaf laid flat to show leaf colour. (D – F) Root systems with nodules removed. Left to right are the control (0 µM copper), 300 µM copper, and 450 µM copper treatments added to basal nutrient solution. G) Nodules attached to roots of the control group. H) Nodules attached to roots of the 450 µM copper group. I) Representative nodules of the control (right) and 450 µM copper (left) treatment groups cut open to show interior colour differences. All bars are 5 mm. (PDF) [file pone.0349086.s003.pdf]

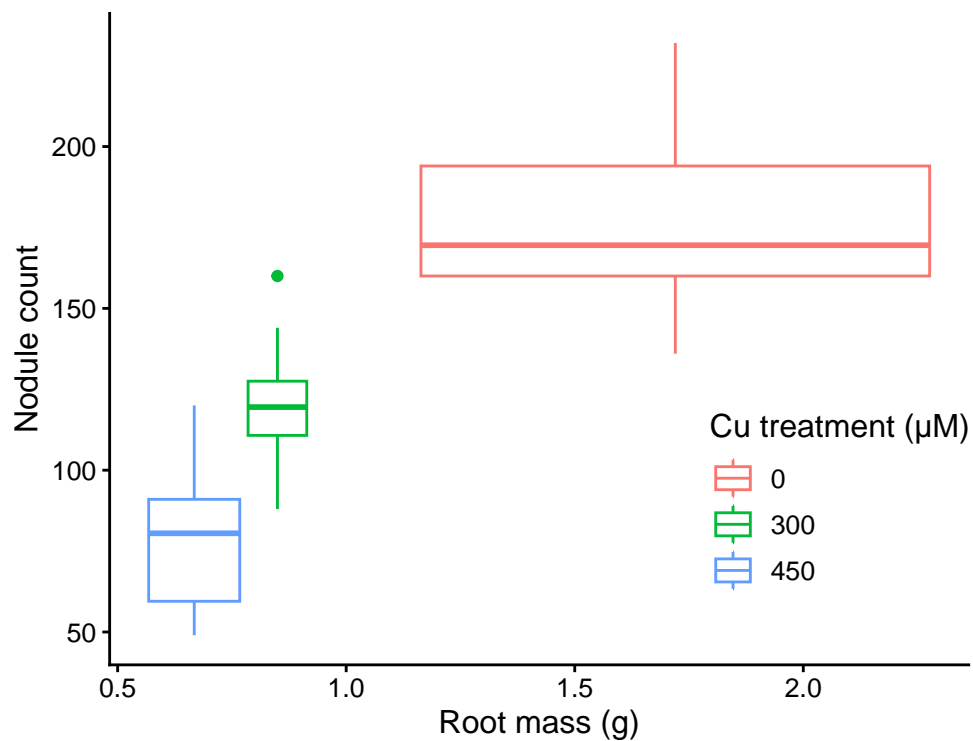

Supplement: S3 Fig — Fresh root mass (g) plotted against nodule number in 0 µM copper (red), 300 µM copper (green) and 450 µM copper (blue) treatments added to basal nutrient solution. (PDF) [file pone.0349086.s004.pdf]

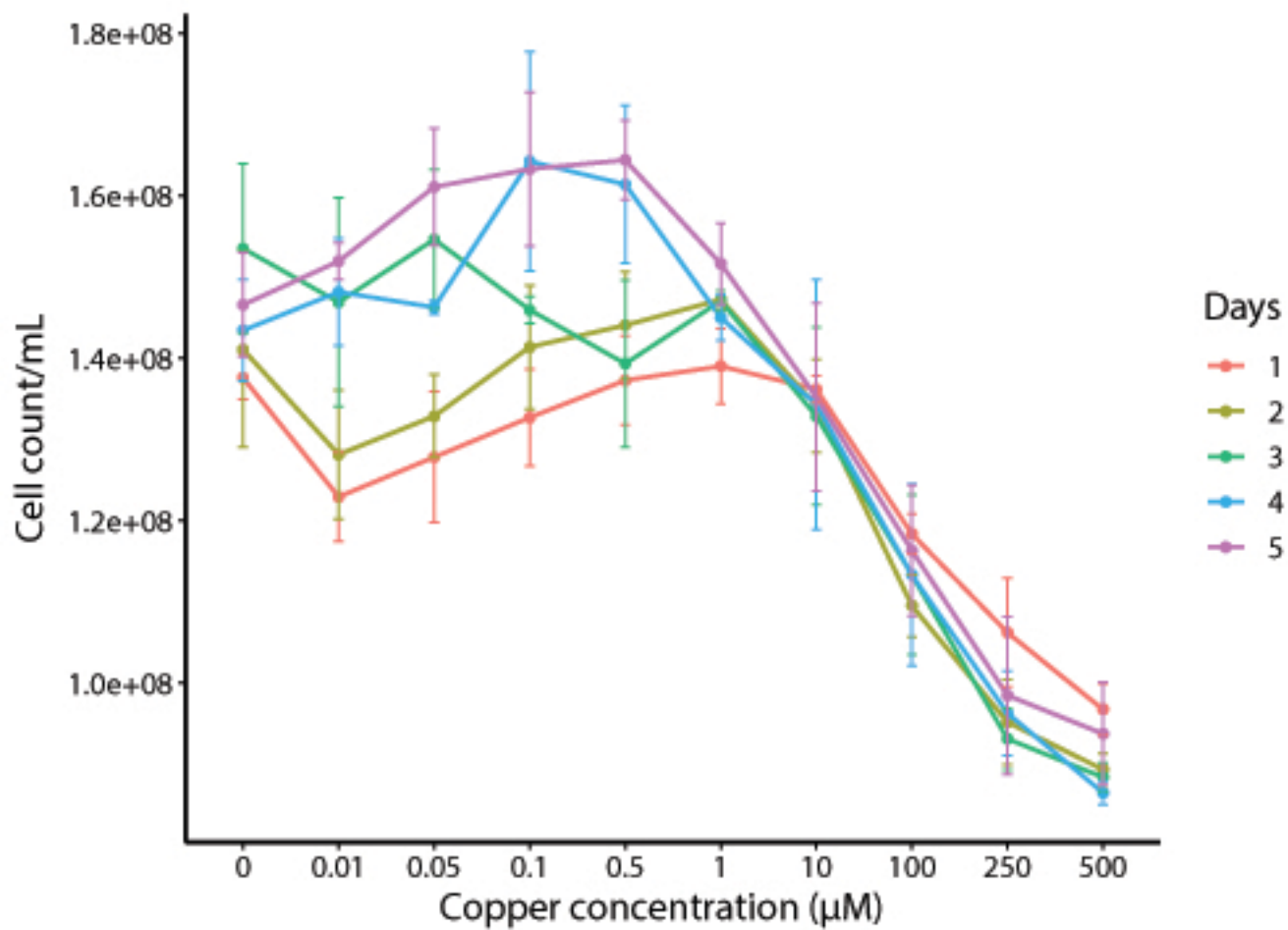

Supplement: S4 Fig — Cells/mL of M. loti at 0–500 µM copper (in addition to the basal concentration of copper in B&D nutrient solution). Data were collected over 5 days (day 0 data not included). Error bars indicate SEM, n = 3. (PDF) [file pone.0349086.s005.pdf]
