# Supplementary material for: Copper causes reduced nitrogen fixation but does not accumulate in the nodules of the legume Lotus japonicus
Source: PLoS One. 2026 May 8;21(5):e0349086. doi: 10.1371/journal.pone.0349086 (PMC13155547; doi:10.1371/journal.pone.0349086)
Supplement: Attachment — Submitted filename: Response to Reviewers.pdf [file pone.0349086.s009.pdf]

Response to Reviewers

Journal Requirements:

1. If the reviewer comments include a recommendation to cite specific previously published works, please review and evaluate these publications to determine whether they are relevant and should be cited. There is no requirement to cite these works unless the editor has indicated otherwise.

Not applicable

2. *Please review your reference list to ensure that it is complete and correct. If you have cited papers that have been retracted, please include the rationale for doing so in the manuscript text, or remove these references and replace them with relevant current references. Any changes to the reference list should be mentioned in the rebuttal letter that accompanies your revised manuscript. If you need to cite a retracted article, indicate the article's retracted status in the References list and also include a citation and full reference for the retraction notice.*

We did not find any retracted papers in our reference list. However, we removed the old reference number 1 because we could not verify its doi . We also removed old reference numbers 9, 17,18, 22, 31, 37, 38 and 53 because they were redundant. We added one new reference (new # 8). The reference list has been checked for completeness, and we have corrected some minor errors.

Reviewer #3

1. In the present manuscript, the authors evaluated the effect of increasing Cu addition on plant growth, nodule activity, and N2 fixation in L. japonicus. The manuscript is very well written. The Materials and Methods section describes in detail the methodologies used in the experiment, as well as the measurement protocols, which is highly appreciated. The statistical analysis is well performed and appropriate for evaluating the effect of the treatments applied.

Thank you for these positive comments.

2. One point of critique is that the manuscript places strong emphasis on oxidative processes occurring within the nodules and on the associated measurements. In my view, the interpretation of the experiment could be simpler. The addition of Cu affected plant growth, likely reducing photosynthesis, which in turn reduced plant N demand and consequently decreased the supply of assimilates to the nodules. This reflects the well-known plant–microsymbiont trade-off (cost–benefit relationship). Importantly, this phenomenon is not only observed under toxicity conditions but also under P deficiency.

Deficiency of other nutrients can also induce nodule senescence, increased leghemoglobin degradation (leading to the green coloration of nodules), and reduced nodule functionality. A decrease in O2 transport to symbiosomes is likely a primary mechanism inhibiting N2 fixation when plant N demand decreases. The observations within the nodules (e.g., reduced leghemoglobin levels) may therefore be consequences of reduced N demand rather than direct oxidative damage. Therefore, this interpretation does not contradict the presented data. I agree with author´s conclusions.

I believe the manuscript would benefit from shortening the arguments related to oxidative processes, particularly since Cu concentration did not increase in nodules. This may represent the main unresolved point of the study: explaining why Cu concentration did not increase in nodules (for example, via qPCR analysis of metal transporters). However, this aspect is partially addressed in the discussion.

Overall, with appropriate refinements to the Introduction, Results, and Discussion sections, the manuscript will present a fully coherent narrative. I agree with the revisions made during previous review rounds and with the authors' responses to reviewers.

We thank the reviewer for these suggestions. In our response below, we have used line numbers from the version with tracked changes.

Introduction:

a. Lines 50-53. We included factors other than oxidative stress that can induce senescence. It now reads: "Rapid early senescence can be induced when the production of sugars by the host plant is limited due to stressors such as defoliation, phosphorus deficiency, darkness and drought [reviewed in 15], or when available N in soil increases [14].

b. We deleted lines 57-58, in which we went into more detail than required about antioxidant enzymes: " Some nodule antioxidant enzymes are reported to upregulate during senescence, while others decrease or do not vary significantly".

c. Lines 73-74. We moved this line from the results section "The presence of Lb in the nodules is closely associated with nodule function because of its role shuttling O_2 to the bacteroids". We placed it in the introduction to build the case for using leghemogloblin concentration as a measure of nodule function.

d. Line 78. We added a line "Leghemoglobin degrades during nodule senescence" to provide more background for our conclusion that our results indicate nodule senescence due to plant stress.

e. lines 81-84. We deleted these because they provided more detail than necessary about antioxidants: "The most abundant non-enzymatic antioxidants of the nodule are ascorbate and glutathione, whose concentrations in the nodule are positively correlated with N_2

fixation [30]. A non-exhaustive list of enzymatic nodule antioxidants includes superoxide dismutase (SOD), catalase (CAT), thiol peroxidases, and the enzymes of the ascorbate-glutathione pathway [26,32,34].

f. Lines 114-119. We deleted information about changes in antioxidant enzyme activity in response to copper: "...activity of SOD in the roots decreased as Cu increased, while activity of APX and CAT increased with Cu until declining at higher Cu treatments [58]. Another experiment on alfalfa showed ROS production in the roots increased when they were exposed to Cu, triggering an increase in the activities of APX, CAT and glutathione reductase at lower Cu concentrations but a decrease in antioxidant activity at higher concentrations [59]." We kept a few words about APX activity to build our case for measuring it in our study.

Results

g. Lines 142-143: We deleted this line because the idea was already in the discussion. Discussion: "Root biomass reduction, particularly fine roots and root hairs, is a characteristic response to excess Cu in many plant species [57, 66]."

h. Lines 196-197. As mentioned in point 3 above, we moved this line to the introduction (lines 73-74): "The presence of Lb in the nodules is closely associated with nodule function because of its role shuttling O_2 to the bacteroids".

i. Lines 220-223: We integrated "This would indicate that while copper was taken up by the plants, it did not accumulate in the nodules in proportion to its availability despite the nodules being immediately adjacent to the roots and having a connected vasculature [68]. This would indicate that Cu transport to the nodule tissues is restricted in some fashion so that internal Cu concentration remains relatively constant" into lines 304-308 of the discussion: "Despite the evidence that excess Cu inhibited nodule development and activity, measurements of tissue Cu concentrations revealed that Cu did not accumulate in the nodule tissues, even though the nodules are immediately adjacent to the roots and have a connected vasculature [72]. This would indicate that Cu transport to the nodule tissues is restricted in some fashion so that internal Cu concentration remains relatively constant".

j. Lines 223-226. We deleted "It is also therefore unlikely that the differences in nodulation and N_2 fixation observed are due to Cu acting directly on the nodule interior" and added "Differences in nodulation and N_2 fixation observed were not due to accumulation of Cu in the nodule interior" to lines 365-366 of the concluding paragraph.

Discussion
See points i and j above.

3. Consider moving some text currently in the Results to the Discussion (lines 133–135, 188–189, 212–218).

Done, see points g toj above.

Response to the editor

1. *To ensure your figures meet our technical requirements, please review our figure guidelines: https://journals.plos.org/plosone/s/figures*

   You may also use PLOS's free figure tool, NAAS, to help you prepare publication quality figures: https://journals.plos.org/plosone/s/figures#loc-tools-for-figure-preparation.

   NAAS will assess whether your figures meet our technical requirements by comparing each figure against our figure specifications.

We checked our figures against the journal's guidelines and submitted them to the NAAS site for evaluation. The NAAS software re-sized the figures to be smaller than the journal's listed figure dimensions by cropping parts of the figures. So, we re-sized the figures to match the output from NAAS. When we re-submitted these smaller figures to NASS, it further reduced the figure dimensions and cropped the margins around each figure, bringing the text of the X and Y axes to the very edge of the field and making them difficult to read. We have opted to submit figures that have slightly smaller dimensions than required by the journal but, to keep the white space (margins) around each figure, do not pass the NAAS assessment. If you require even smaller figures, we would be happy to provide them.
