# Supplementary material for: Copper causes reduced nitrogen fixation but does not accumulate in the nodules of the legume Lotus japonicus
Source: PLoS One. 2026 May 8;21(5):e0349086. doi: 10.1371/journal.pone.0349086 (PMC13155547; doi:10.1371/journal.pone.0349086)
Supplement: Attachment — Submitted filename: Response to Reviewers.docx [file pone.0349086.s007.docx]

**Response to Reviewers**

Response to academic editor:

1. *Please ensure that your manuscript meets PLOS ONE's style requirements, including those for file naming.*

We made several changes to better align with the guidelines provided by PLOS: we edited file names for figures and supporting information, changed the font of entire document to Times New Roman, centered the title on the first page, changed all equations that appear in the manuscript to be numbered and inserted via Word’s Equation Tool, and moved the Supporting Information captions to follow the references.

*2. We note that there is identifying data in the Supporting Information file <S1 Table.docx>. Due to the inclusion of these potentially identifying data, we have removed this file from your file inventory.*

The only identifying data we could find in the Supporting Information files were authors’ names in the metadata, so we have removed these names and changed all supplementary figures to an anonymous file format (PDF with no author). We are not sure that the anonymization process worked and would appreciate it if PLOS would check that all identifying information was removed.

Responses to Reviewer 1:

*1. Line 32 - the ability to fix nitrogen should be clarified as being characteristic of bacteroids, not plants*

We edited line 33 of the version with tracked changes to clarify this point. The new sentence is: “Legumes are an important source of usable nitrogen (N) in terrestrial ecosystems because of their symbiotic association with rhizobia that fix atmospheric N_2_ to ammonia [1].”

*2. Results - the results regarding the determination of MDA and APX for shoots are missing, as these results would provide a broader, more accurate answer to the statement in the title of the paper.*

We agree that shoot APX and MDA content would be beneficial in understanding the whole-plant response to Cu. However, because we initially found that Cu accumulation in response to Cu treatment was highest in the roots and nodules and less than 10% of the total Cu in the plant was found in the shoots (Fig 1), we chose to focus our experiments on the below-ground tissues. We believe that our focus primarily on the nodules is appropriately reflected in the article title: “Copper-induced stress in the legume *Lotus japonicus* causes loss of nitrogen fixation but not oxidative damage **in nodules**”.

*3. Please expand abbreviations used for the first time, especially for reagents, buffers, etc., e.g., PPB.*

Potassium phosphate buffer was defined in the Methods section “Leghemoglobin concentration” in line 426 in version with tracked changes, which is the first time it was used. We could not find any undefined terms.

*4. Please add the equipment used for analyses.*

All the equipment/materials/settings used in the experiments are now specified in the methods section with more detail. We found that several papers published by PLOS ONE also include the make/model of their equipment, so these details have also been included. The text in the relevant methods sections has been edited for clarity after these additions.

Responses to reviewer 2:

*1. Considering that the negative effects of Cu on the RNS has been widely documented, the relevance of this study is to explore at molecular level, the direct or indirect effect of this heavy metal.*

We thank the reviewer for appreciating the value of our study.

*2. Cu treatment might interfere with the early steps of the legume-rhizobia symbiosis. For instance, what happens to root hair growth in Cu-treated plants? Lotus plants with compromised root hair growth/development are affected in IT and nodule formation. What´s the effect of Cu treatment in root hair-IT formation? IT counting is a standardized procedure to answer this question in L. japonicus.*
We agree that a study of root hairs and infection threads would provide valuable additional information. We did measure the mass, number and function of nodules in our study, with the results indicating that the concentrations of Cu we used did not prevent the formation of infection threads. We are not in a position to do another study to measure root hairs and infection threads in Cu-stressed plants, but we did address the importance of these variables in lines 274-279 of the version with tracked changes: “The tips of emerging root hairs are the primary sites of entry for rhizobia and are important for the formation of infection threads [70,71]. Reduced nodulation was observed in *L. japonicus* mutants that produced no root hairs (*rhl1* and *slp*) [72], mutants with altered biosynthesis pathways critical for root hair development (*dahps1-1* and *dahps1-2*) [73], and plants with thicker and shorter root systems (*rdol*) [74]. Root biomass reduction, particularly fine roots and root hairs, is a characteristic response to excess Cu in many plant species [57,64].”

*3. The ARA calculated per nodule weight indicates that nodule functioning is directly affected by Cu-treatment. In this regard, additional information could confirm or contradict this hypothesis. How is nodule occupancy by rhizobia? Or the expression of nitrogen-fixing genes?*

We do not have data for rhizobial count/nodule occupancy. But the purpose of counting the nodules, using the ARA for nitrogenase activity and the measurements of nodule leghemoglobin were ultimately to assess nodulation and rhizobial function within those nodules. We believe this sufficiently shows the effect of Cu in these tissues. Expression of relevant genes would be very interesting and we hope future research will include this direction of inquiry, but we are not in a position to do these analyses.

*4. The phenotype of plants should be included in the main figures, along with the plots.*

We appreciate the reviewer’s desire to see both images and data of the plant phenotypes together, presumably in Fig 1. Representative photographs are available in the supplementary files (S2 Fig); however, we believe combining these figures would make the final image very large, becoming a 12-panel figure. If the editor would prefer, we could combine Fig 1 and S2 Fig into a single figure, or move S2 Fig into the main manuscript; we are willing to do so.

*5. The number of biological replicates and total number of harvested plants is not clear in Material and Methods and figure legends. / Legend of Figure 1. It is not clear the number of tested/harvested plants and the number of biological replicates. It is written n=5 to n=14, depending on the panel. Five plants do not seem a robust number for solid conclusions, considering that L. japonicus is a small plant that allows high number of plants under laboratory conditions.*

We edited the second paragraph of the Materials and Methods section (lines 388-391 in the version with tracked changes) to better explain the number of plants harvested vs. biological replicates, and what each datapoint represents. The new text is: “Because the final number of plants in each pot varied from 7 to 8, the average tissue mass, length, and nodule count of the plants in each pot was calculated. The average from each pot was then considered an independent biological replicate”.

All figure captions were also edited for clarity about the number of replicates used in each experiment. We hope the new Materials and Methods explanation makes it clearer that each replicate is not a single plant but the average of a group of plants grown in the same pot.

Some measurements, like root mass, were taken at the beginning of all experiments and so have a greater number of replicates.

*6. Since Cu treatment have a negative impact on the root mass, this might explain the reduced nodule numbers as well. Plotting the nodule numbers by root mass/length would be very informative.*

We agree that the reduced nodule number/mass is linked to the reduced root mass due to Cu exposure, and we addressed this in the discussion section (lines 279-281 in the version with tracked changes): “The Cu-induced reduction in root mass seen in our experiment [S3 Fig] contributed to the drop in nodulation, an observation shared by other researchers [5,63].” Because our root mass and nodule count data are already shown in Fig 1, we have not duplicated the data in a new figure in the main body of text. Instead, we have added a supplementary figure (S3 Fig) in which nodule number is plotted against root mass to further illustrate this relationship for readers.

*7. Figure 3. Having representative images of nodules and nodule sections would be very informative.*

We have added representative photos of nodules and nodule cross-sections from the 0 µM Cu and 400 µM Cu groups to S2 Fig. They were originally omitted due to poor picture quality.

*8. Please include the age plant, and days after inoculation in the figure captions.*

Figure captions have been edited to include age of plants and days after inoculation that they were harvested.

*9. Readability can be improved with composite figures. For instance, Fig 2 and Fig 3 are related to nodule functioning and could be merged into a single figure.*

We agree with the reviewer. We combined old Fig 2 and old Fig 3 into a single composite figure (new Fig 2). As Reviewer 2 stated, they are both ultimately measurements of nodule function and so it was appropriate to present them together. Edits were made to the caption and in-text referrals to the figure to accommodate this change. Old Fig 5 and old Fig 6 were also combined into a single composite figure (new Fig 4) as they are both measurements related to oxidative stress in the nodules and root systems. Edits were made to the caption and in-text referrals to the figure to accommodate this change.

*10. Line 256. “Reduced nodulation was observed in L. japonicus…”. Ljdahps1 mutants are also defective in root hair development and nodulation.*

We appreciate this information from reviewer 2, which provides another example of reduced root growth impairing nodulation. We have included a reference on *dahps1* mutants in our discussion: “Reduced nodulation was observed in *L. japonicus* mutants that produced no root hairs (*rhl1* and *slp*)[72], mutants with altered biosynthesis pathways critical for root hair development (*dahps1-1* and *dahps1-2*)[73], and plants with thicker and shorter root systems (*rdol*) [74]” (lines 275-278 in version with tracked changes) and edited the remaining reference numbers to accommodate this change.

*11. Line 269. “Total N2 fixation in our experiment was reduced due to the Cu-induced decrease in the number…” Should be highlighted that N2 Fixation was also significantly lower when is calculated by nodule weight. This should be discussed as well.*

We have edited line 290 in the version with tracked changes to highlight that both number and biomass of nodules are being considered when discussing the reduction in N_2_ fixation. We note that this section already makes specific mention of the effect of nodule mass on N_2_-fixation: “Total N_2_ fixation in our experiment was reduced due to the Cu-induced decrease in **both the number and biomass of nodules formed,** and may also have been directly affected by a drop in nitrogenase activity within the nodules themselves. **As nitrogenase occurs exclusively in the bacteroids within the plant’s nodules, lower nodule mass naturally results in a reduction in the total nitrogenase activity of the plant.**”
