# Supplementary material for: Copper causes reduced nitrogen fixation but does not accumulate in the nodules of the legume Lotus japonicus
Source: PLoS One. 2026 May 8;21(5):e0349086. doi: 10.1371/journal.pone.0349086 (PMC13155547; doi:10.1371/journal.pone.0349086)
Supplement: Attachment — Submitted filename: Response to Reviewers .docx [file pone.0349086.s008.docx]

Response to Reviewers

The line numbers mentioned in this list refer to those in the file named “Revised Manuscript with Track Changes”.

1. *Unfortunately, the manuscript submitted for review failed to confirm the claim included in the title.*

We have edited the title to better reflect the claims we make in our paper. We believe the new title, “Copper causes reduced nitrogen fixation but does not accumulate in the nodules of the legume *Lotus japonicus*” is fully supported by our data.

2. *Only MDA and APX activity levels were determined as a reflection of oxidative stress levels – unfortunately, this is not enough to draw conclusions based on these two parameters alone.*

We agree with the reviewer’s point that testing a broader array of antioxidants is required for definitive conclusions about the plant and nodule oxidative stress response. We address this briefly in the discussion section (lines 367-370: “No studies could be found that measured APX or any other antioxidant in the nodule in response to Cu specifically, and the narrow scope of antioxidant responses tested in this study mean that definitive conclusions about the nodule redox state cannot be drawn without further research.” and lines 378-380: “…our study did not capture a wide enough range of antioxidant activity for a fulsome picture of antioxidant responses to Cu-induced ROS in the roots and nodules”.

We have also made a minor edit to the results section (lines 271-272 were deleted remove the suggestion that our data can be used to indicate antioxidant activity.

a. *There is no description of the Lotus nodule type (including an anatomy and cortical tissues) and the aging process specific to this type.*

We have added some information about determinate and indeterminate nodule morphologies (lines 44-48: “Nodules primarily fall under two morphologies: the indeterminate and determinate nodule [12, 13]. Indeterminate nodules are characterized by persistent meristematic activity and create cylindrical nodules with specific zones of activity. Determinate nodules (such as those seen in *L. japonicus*) lose meristematic activity after rhizobial infection and an initial period of cell division, forming spherical nodules with fairly homogenous inner tissue [9,12,14].)

We also added information about their different aging/senescence processes (lines 49-63: “Nodule senescence is a regulated process that can be considered the final step in the lifespan of the nodule [9,15,16]. N_2_ fixation activity peaks at approximately 4 weeks, after which activity steadily declines [15,16]. Symbiosome membrane disintegration begins and lytic symbiosome compartments are formed in order to facilitate nutrient remobilization and reabsorption processes, ultimately ending in the death of the host cell [9,17]. In unstressed nodules, cells senesce sequentially from oldest to youngest, meaning that in indeterminate nodules it progresses from zone IV outwards, and progresses radially from the center in determinate nodules [11,16]. In determinate nodules, bacteroids do not terminally differentiate and are released during senescence, able to revert to a free-living lifestyle [9,11]. Stressors like defoliation, dark, and drought can induce rapid early senescence, as can available N in soil [9,17,18]. Senescence is generally characterized by activation of proteolytic enzymes, a decrease in cytosolic proteins [11,17], and by shifts to the nodule redox balance due to the formation of reactive oxygen species (ROS) and changing activity of nodule antioxidants [17,19]. Some nodule antioxidant enzymes are reported to upregulate during senescence, while others decrease or do not vary significantly [15].”).

We do not address nodule internal anatomy in detail, as we did not separate the internal tissues of the nodules for any experiments, and cannot make any conclusions specific to a nodule tissue type.

b. *The information about antioxidative system in Cu-treated plants and nodules is also missing.*

We have added additional information on nodule antioxidants to two places in the introduction section. First, to the paragraph concerning plant and nodule antioxidant systems (lines 83-89: “Nodule antioxidants are similar to those found in the rest of the plant but are present at higher concentrations to compensate for O_2_ produced during the highly energetic process of N_2_ fixation [30,31]. The most abundant non-enzymatic antioxidants of the nodule are ascorbate and glutathione, whose concentrations in the nodule are positively correlated with N_2_ fixation [30]. A non-exhaustive list of enzymatic nodule antioxidants includes SOD, catalase (CAT), thiol peroxidases, and the enzymes of the ascorbate-glutathione pathway [26,32-34].”).

Second, to the paragraph about copper toxicity in plants (lines 128-137: “Alfalfa seedlings exposed to 0 to 400 mg/kg Cu had reduced biomass, tissue N concentrations, and nodule number and weight at higher Cu concentrations. The antioxidant response to Cu-induced ROS is more variable; activity of SOD in the roots decreased as Cu increased, while activity of APX and CAT increased with Cu until declining at higher Cu treatments [58]. Another experiment on alfalfa showed ROS production in the roots increased when they were exposed to Cu, triggering an increase in the activities of APX, CAT and glutathione reductase at lower Cu concentrations but a decrease in antioxidant activity at higher concentrations [59]. Soybean grown in 0 to 100 µM Cu showed reduced nodule number and size, and a similar dose-dependent response in antioxidants in response to Cu [60].”).

We hope this elaborates sufficiently on antioxidant systems in the nodule throughout its lifecycle and clarifies the specific impact of copper on nodule function and nodular antioxidant systems.

4. *line 46 - "between photosynthate production" should be “between the level of photosynthesis and”*

We have made edits to the lines 67-69, which now read: “…new nodule formation must be regulated to maintain equilibrium between energy production via photosynthesis in the shoots and energy consumption in the nodules [14].”

5. *line 53 - remove "phytoglobin"*

The suggested edit has been made (line 75).

6. *lines 70-71 - "Membrane disintegration, release of the bacteroid from the symbiosome" ........., This description is Unclear. Please clarify the process of symbiosome degradation in the determinate nodule, as this is the case with Lotus.*

This section (lines 49-63) has been expanded to better explain the stages of senescence, and the unique characteristics of determinate nodules in particular (see the second paragraph of our response to point 3a).

7. *Line 79, 80 - correct the notation of units throughout the text.*

Notation of units have been edited throughout the text (e.g., from mg⋅kg^-1^ to mg/kg) to conform to unit styles we observed most frequently in other PLOS One papers.

8. *Please expand the abbreviations used for the first time: MDA, PPB.*

All abbreviations are defined in the text at their first use.

a. *The results suggest that Cu does not directly affect activity within the nodule through oxidative damage. However, Cu exposure did not demonstrate increased APX activity in the nodule, which the authors explain as "indicating a loss of antioxidant activity in the nodule in Cu-treated plants," although the results indicate that Cu is not accumulated in the nodules.*

We believe that the last sentence of our results section may have misled the reviewer. As the reviewer states, our results indicate that Cu does not accumulate in the nodules and does not directly affect nodule activity. We found no evidence of lipid peroxidation in nodules with increased Cu treatment, further indicating that Cu is not causing oxidative damage directly to the nodules. We have edited the last sentence of the results section to focus solely on the numerical response to experimental treatment. We have also elaborated on our explanation of Cu-induced changes in APX activity in the discussion section (lines 357-380). We suggest that reduced APX activity in nodules is due to Cu stress in the roots and shoots of the plant, in turn inducing premature senescence of the nodules.

b. *It can therefore be assumed that other components of the antioxidant system participate in reducing stress levels in the nodules, but not APX. However, the study did not demonstrate the level of the antioxidant system in the nodules what would be key in this topic. Therefore, the manuscript cannot be accepted in its current form.*

We agree with the reviewer that a complete antioxidant profile would allow us to draw more concrete conclusions about the response to Cu-induced ROS in the entire plant, including nodules, and have addressed this criticism in point 2 above. It is very likely that antioxidants that were not measured in our study are active. However, we believe the assumption that other nodule antioxidants must be the key to the nodule’s response is incorrect. The lack of Cu accumulation or measurable oxidative damage in the nodules themselves strongly supports our primary finding that Cu’s effect on N fixation is not due to a direct effect of Cu-induced oxidation in the nodules.

Additional changes:

We have made some changes to the order of paragraphs in the introduction, particularly the paragraph about nodule senescence. Along with the edits to the paragraph contents, we believe this change increases the readability and overall flow of the introduction section.

We made appropriate edits to the in-text citations and references section in accordance with edits made to the text.

We have removed our list of abbreviations to conform to the style we observed most frequently in other PLOS One papers.
